# Technological advances for analyzing the content of organ-on-a-chip by mass spectrometry

**DOI:** 10.3389/fbioe.2023.1197760

**Published:** 2023-05-22

**Authors:** Darya Hadavi, Ilona Tosheva, Tiffany Porta Siegel, Eva Cuypers, Maarten Honing

**Affiliations:** Maastricht Multimodal Molecular Imaging (M4i) Institute, Division of Imaging Mass Spectrometry Maastricht University, Maastricht, Netherlands

**Keywords:** organ-on-a-chip, mass spectromelry, real-time analyis, online analysis, analytical technique, interface

## Abstract

Three-dimensional (3D) cell cultures, including organ-on-a-chip (OOC) devices, offer the possibility to mimic human physiology conditions better than 2D models. The organ-on-a-chip devices have a wide range of applications, including mechanical studies, functional validation, and toxicology investigations. Despite many advances in this field, the major challenge with the use of organ-on-a-chips relies on the lack of online analysis methods preventing the real-time observation of cultured cells. Mass spectrometry is a promising analytical technique for real-time analysis of cell excretes from organ-on-a-chip models. This is due to its high sensitivity, selectivity, and ability to tentatively identify a large variety of unknown compounds, ranging from metabolites, lipids, and peptides to proteins. However, the hyphenation of organ-on-a-chip with MS is largely hampered by the nature of the media used, and the presence of nonvolatile buffers. This in turn stalls the straightforward and online connection of organ-on-a-chip outlet to MS. To overcome this challenge, multiple advances have been made to pre-treat samples right after organ-on-a-chip and just before MS. In this review, we summarised these technological advances and exhaustively evaluated their benefits and shortcomings for successful hyphenation of organ-on-a-chip with MS.

## Introduction

The use of three-dimensional (3D) cell cultures has been growing due to their widespread application ranging from studying drug efficacy and toxicity to creating disease models (Zheng et al., 2016). By using a 3D-culture model, one can mimic the *in vivo* environment of human physiology more accurately than two-dimensional (2D) models, such as standard cell culture (Lee and Sung, 2018; Mittal et al., 2019). Therefore, the quality of the conducted experiments improves. The progress of 3D devices and technologies has advanced to the development of microfluidic chips to capture organ-level function known as organ-on-a-chip (OOC) (Zhang and Radisic, 2017; Zhang et al., 2018). The design of the OOC devices is made to mimic the human cellular microenvironment. It includes the flow of fluids through micro-channels to mimic the vasculature network for providing nutrients and transporting waste and metabolites. The OOC models could also simulate the physical environment of human organs (e.g., lung, gut, and kidney) by mimicking the structural features (Caplin et al., 2015; Sosa-Hernández et al., 2018; Wu et al., 2020). This can eventually facilitate the translation of the *in vitro* findings to the human condition.

The use of OOCs has the potential to make the drug discovery process fast and cheaper (with a cost reduction of up to 26%) (Franzen et al., 2019). However, despite many advances, its applicability is hampered by the lack of online detection schemes, allowing the real-time observation of cellular behaviors (An et al., 2015; Probst et al., 2018). This shortage limits our understanding of cellular mechanism as a function of time and consequently unable us from correcting for the defects in the produced OOC models. The analytical techniques that have been used so far include optical imaging, electrochemical sensors, fluorescence- or label-free assays such as photonic crystal in a total internal reflection (Morales et al., 2016), capillary electrophoresis, and mass spectrometry (MS) (Wikswo et al., 2013).

Among these techniques, MS offers high sensitivity and specificity to analyze changes in metabolites, proteins, and lipids (Caballero et al., 2003; Glish and Vachet, 2003; Zhou and Veenstra, 2008). Utilizing MS for the *in-situ* monitoring of 3D cell systems in OOC can provide insight into the molecular composition of culture media, excreted metabolites, and waste products (Ahadian et al., 2018). Despite the multiple advantages of MS, it cannot be directly coupled with OOC for online and real-time analysis of molecules of interest (e.g., cytokines, proteins, chemokines) (An et al., 2015). This problem occurs mainly due to the presence of cell media in the chambers of OOC, which is rich in salts, nonvolatile buffers, and compounds that can hamper the MS analysis by creating ion suppression (Wikswo et al., 2013). Currently, offline sample preparation methods are used to normalise chip-to-chip variability or manipulate cell excretes before MS analysis (Gallagher et al., 2023). However, with these approaches, the time-resolved detection of metabolites is largely reduced, which is an essential factor for unravelling cellular mechanisms.

Considering the numerous advantages offered by MS for biomedical applications, this paper focuses mainly on the investigated approaches for direct coupling of OOC with MS. The capabilities and weaknesses of each approach for real-time analysis are reviewed in detail.

The review begins with an introduction to OOC and used analytical techniques to evaluate the mimetic tissue models. After a brief discussion on the techniques of phase contrast microscopy, enzyme-linked immunosorbent assays (ELISA), transepithelial electrical resistance (TEER), the review is mainly focused on MS. To this end, MS introduction is followed by hyphenation techniques that bridge OOC with MS, namely, electrophoresis, solid phase extraction, liquid chromatography and droplet-based chips, and their limitations are discussed. This article provides an exhaustive review of relatively new developments that would potentially enable the development of a robust and reliable interface for analyzing OOC content with MS as a rapid, sensitive, and specific analytical technique.

## Introduction to organ-on-a-chip

**FIGURE 1:**
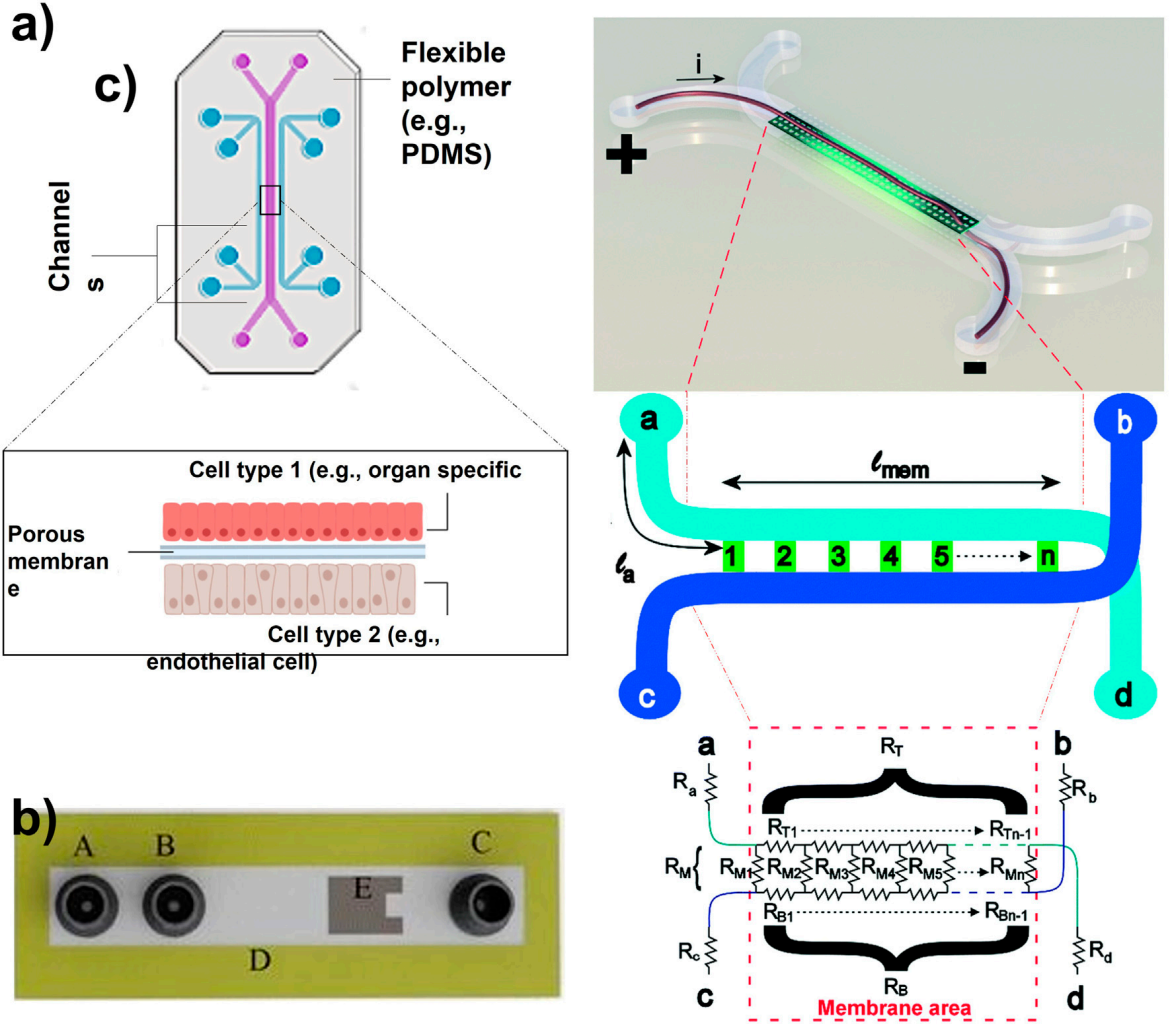
**(A)** Organ-on-a-chip with two parallel channels separated by a porous membrane (Adapted from Zhang et al. (Zhang et al., 2018),.). **(B)** Schematic of a microfluidic device with integrated thin-film thermopile. A is the first inlet to the supply buffer solution, B is the second inlet to supply the analyte, C is outlet/waste, D is the channel wall, and E is the thermopile to measure the heat of the enzymatic reaction (Adapted from Nestorova et al. (Nestorova et al., 2015),.). **(C)** Artist impression of the organ-on-a-chip with integrated TEER with simplified geometry of the chip. The circuit model presents the working principle of the chip. The inlet and outlet channels la to ld are shown by resistors Ra to Rd. Channels a–d and b–c are connected by the membrane and indicated by lmem (the length of the membrane) and the red dashed square [Adapted from Odijk et al. (Odijk et al., 2015)].

Organ-on-a-chip devices simulate human micro-physiological systems, which is a powerful alternative to conventional 2D *in vitro* testing (Lee and Sung, 2018; Probst et al., 2018; Mittal et al., 2019; Rothbauer et al., 2019). The OOC field emerged almost 25 years ago starting with microfluidic-associated microfabrication techniques and moving toward more physiologically relevant cell cultures (Junaid et al., 2017). The common composition of an OOC is a flexible polymer the size of a computer USB stick, that contains microfluidic channels lined by living human organ-specific cells, interconnected with human endothelial artificial vasculature (Figure 1A) (Lee and Sung, 2013). This design provides the scientist with a window into the inner working condition of human cells in living tissues. Consequently, it allows them to study the molecular- and cellular-scale activities that drive human organ function (Lee and Sung, 2013). The OOC is at the Frontier of microfluidics, tissue engineering, and stem cell biology (Skardal et al., 2017; Rothbauer et al., 2019). As OOC devices attempt to replicate human physiology, they have been implemented in mechanical studies as well as functional validation (Huh et al., 2010). Furthermore, they can be potentially implemented in molecular pharmacology testing during the drug discovery phase, giving information on the mode of action, efficacy, and toxicity of the drug candidates in lead libraries (Peel et al., 2019). The fast advances in this field can not only decrease the costs of pharmacological studies (Bhise et al., 2014) but possibly enables the testing of drug combinations at different concentration levels facilitating the design of treatments in personalized medicine. For example, cancer patients have different responses to the given treatment. The use of OOC for the growth and observation of patient-specific cells can assess the most convenient treatment and drug concentrations for each patient (Caballero et al., 2017). Additionally, OOCs can also be used as neural-systems-on-a-chip for target-based or phenotypic screenings using patient-specific disease models, establishing highly effective treatments (Haring et al., 2017).

Thus far many OOCs have been developed, including specific conditions for the organ or tissue of interest. These conditions include pressure, flow rate, pH, osmotic pressure, nutrient content, and toxins’ presence or absence (Mittal et al., 2019). Amongst the many OOCs, one can find lung-, liver-, kidney-, gut-, skin-, brain-, heart- and even placenta-on-a-chip (Caballero et al., 2017; Sosa-Hernández et al., 2018). With the studies conducted in the past years, researchers are aiming to bring this field a step further by developing a human-on-a-chip, leading to mimicking the whole-body physiology in multiple connected OOCs (Lee and Sung, 2013; Zhang and Radisic, 2017; Wu et al., 2020; Ingber, 2022; Driver and Mishra, 2023). Therefore, this would allow for the observation and analysis of how different agents influence the physiological functioning of the body as a whole. Eventually, it can also allow for progress in personalized medicine (Bhise et al., 2014; Zhang et al., 2018), prediction of quantitative pharmacokinetic parameters (Herland et al., 2020), and examination of metastasis processes by metastasis-on-a-chip devices (Caballero et al., 2017).

With this goal to simulate the physiological environment of human organs as accurately as possible (Wu et al., 2020), OOC devices are widely claimed as a potential tool in replacing animal studies (Van Der Meer and Van Den Berg, 2012). However, with current knowledge, this replacement seems very challenging. One of the obstacles in this transition is the lack of analytical technologies, able to selectively detect low concentrations of a large diversity of molecules in a time-resolved manner, and both in or outside the cells. It seems obvious that the availability of real-time measurements will serve to monitor the dynamic behavior of the cellular microenvironment. In the upcoming sections, the analysis method of OOC is evaluated.

## Organ-on-a-chip analytical techniques

Some of the challenges that come along with the implementation of OOCs in research include the analytical techniques that have been commonly used for chip analysis (An et al., 2015; Probst et al., 2018). Evaluating the tissue behavior in OOC devices requires accurate, non-invasive, and real-time measurement of cell functions (Ahadian et al., 2018). Some of the analytical techniques that have been implemented for on-/off-chip analysis include phase contrast microscopy, ELISA, TEER and MS compared in Table 1 (Wikswo et al., 2013; Junaid et al., 2017).

**TABLE 1:** Organ-on-a-chip analytical techniques and their shortcomings.

Organ-on-a-chip analytical techniques	Shortcomings
Optical imaging	Low field of view at high magnifications
	Low working distance and fabrication challenges
	Suitable for static analysis
Fluorescent microscopy	Fluorescent labelling is required
Confocal microscopy	Phototoxicity due to laser intensity
Thermoelectrical ELISA	Heat loss
	Decreased sensitivity and received signal magnitude
TEER	Difficulties in integrating electrodes on the OOC
	Electrode displacement influences results
MS	Sample pre-treatment is required

### Microscopy

Various optical imaging techniques have been used to monitor OOC platforms. Amongst them are, bright-field microscopy, phase contrast microscopy, and fluorescent and confocal microscopy (Arandian et al., 2019). To monitor the cellular level activities in an OOC by optical microscopies, high magnifications (e.g., 400x—1500x) and a resolution of 0.25 µm are preferred. One of the general disadvantages under these conditions is related to a low field of view at high magnifications and resolutions (Paiè et al., 2018; Arandian et al., 2019). The limited field of view obscures observing the total area of micron-sized chips. This limitation becomes more concerning when dynamic cellular mechanisms are under investigation. Another aspect to consider when utilizing microscopes is the required low working distance between the objective lens and the surface of the sample for better resolution and magnification (Arandian et al., 2019). This requirement imposes the fabrication challenge to OOC devices, which must have a similar thickness and refractive index to those of the used objectives. This in turn dramatically restricts the choice of material for chip manufacturing and inhibits the fabrication of complex designs. Additionally, the use of either fluorescent or confocal microscopy in OOC analysis faces the researchers with challenge of attaching fluorescent molecules to the appropriate biological agents (Arandian et al., 2019). Furthermore, although confocal microscopy is a technique of high quality for the analysis of OOC, the intensity of the laser beam should be carefully controlled to prevent phototoxicity of the cells on the chip, thus the optical setup has to be highly precise (Arandian et al., 2019).

### Enzyme-linked immunosorbent assays (ELISA)

ELISA enables measuring the enzymatic activity of analytes, antigens, and antibodies. The process is based on the application of enzymes as labels and the subsequent detection of the occurring enzymatic reactions (Premjeet et al., 2011; Nestorova et al., 2015). Nestorova and others presented the use of thermoelectric direct sandwich ELISA as an analytical technique for OOC (Figure 1B). Their microfluidic device included a channel wall with immobilized primary antibody and an inlet to introduce unmodified analyte. The same inlet was used to supply enzyme-linked reporter to form primary antibody-analyte-reporter antibody complex. A separate inlet was used to provide a laminar flow of substrate for the enzymatic reaction. This reaction releases heat which was detected by a thermopile sensor implemented in the microfluidic device. This relatively new approach relies on the determination of the analyte concentration based on the produced heat from the enzymatic reaction between the substrate and the enzyme-linked reporter. Although successful, this approach hides the down point of heat loss, thus decreasing the sensitivity and the magnitude of the signal received from the OOC (Nestorova et al., 2015). Generally, one of the main concerns regarding biosensors is related to the risk of detecting non-specific proteins due to their overabundance compared to the analytes of interest.

### Transepithelial electrical resistance (TEER)

TEER is an electrochemical sensing technique, which measures the barrier integrity of epithelial and endothelial layers (Santbergen et al., 2019). This approach is usually used as an analytical technique for disease models, or as a toxicology marker. The TEER measurements are non-invasive and real-time. TEER design consists of submerged electrodes in both the top and bottom compartments of the *in vitro* transwell system (Santbergen et al., 2019). Odijk *et al.* tried to implement TEER in OOC analysis, which revealed that the obtained results from OOC are comparable to transwell systems (Figure 1C) (Odijk et al., 2015). Henry *et al.* also implemented TEER electrodes on an OOC by patterning them on a polycarbonate substrate (Henry et al., 2017). The research group explained that their system provided sufficient sensitivity and enabled real-time measurements. However, the location, dimensions, and design of the electrodes could be improved. The slightest displacement of the electrode can significantly impact the TEER results, causing variation in measurements (Odijk et al., 2015). Generally, the integration of these electrodes in the closed areas of an OOC device is risky due to the smaller cell culture area of OOC compared to transwell systems. Therefore, this complexity makes the fabrication and reliability of TEER on OOCs more challenging.

Taking to account the shortcomings of the mentioned analytical techniques, to increase the translational relevance of OOC in a research setting, quantitative analytical techniques offering online and real-time analysis are still missing. Mass spectrometry offers multiple advantages in this regard.

### Mass spectrometry (MS)

Mass spectrometry is one of the most used analytical tools that offer multiple advantages for OOC analysis (Chen et al., 2021). When analyzing the cellular excretes from the 3D cellular environment of an OOC, a diverse range of molecules are encountered. Some of these molecules are already known material through decades of research on cellular mechanisms. However, the quantity of these materials might change to different pathological conditions. Analyzing OOC content by MS enables quantifying these known compounds and comparing healthy *versus* disease models. On the other hand, not all cellular mechanisms are elucidated yet. One step to unraveling cellular behaviors is to identify the involved unknown compounds. Here again, MS offers the capacity to identify unknown molecules with high sensitivity and confidence. In addition, rapid detection of molecules by MS facilitates online, real-time, and high throughput screenings. Hence, the hyphenation of OOC with MS can assist in understanding the cellular behavior and reaction pathways as well as optimizing an OOC device to be a better representative of natural human physiology. One main concern in connecting OOC with MS is related to matrix effect that can influence sensitivity and selectivity, subsequently the precision, accuracy, and robustness of results (Panuwet et al., 2016). The matrix effect mainly happens when analysing biological matrices. This effect is caused by either suppression or enhancement of ionization efficiency of target analytes due to the presence of matrix compounds. In the case of analysing OOC content, usually the used cell media include non-volatile compounds, causing matrix effect. Hence, direct connection of OOC to MS requires an interface to reduce this effect before MS analysis.

## Introduction to mass spectrometry

Every MS instrument is composed of three main components including–an ion source for the ionization of samples, a mass analyzer for the separation of ions (i.e., based on mass-to-charge (*m/z*)), and a detector (Awad et al., 2015). The MS devices measure primarily the mass-to-charge ratio of electrically charged ions providing information about the elemental composition of compounds (Choo and Tham, 2007; El-Aneed et al., 2009). Thus, the more accurate the *m/z* is, the more confidence there is about the elemental composition of the tested composite. In addition, MS instruments can be used to fragment the precursor ions to generate products or fragment ions. The so-called tandem mass spectrometry (MS/MS), provides structural information on the compound and enables identification (SUSlick, 1998).

MS has been coupled with various interfaces such as liquid chromatography (LC-MS), and ion mobility spectrometry (IMS-MS) (Hadavi et al., 2019; Hadavi et al., 2022a; Hadavi et al., 2022b), expanding the applicability of this analytical tool. Currently, the LC-MS(/MS) is a widely used technology as it enables the separation and sensitive detection of a wide range of molecules, including potential isomers (Holčapek et al., 2012; Theodoridis et al., 2012). LC allows for online clean-up of the sample, often a complex biological matrix, and thus opens possibilities to clean up salts and reduce chemical background. Ion mobility MS is a gas-phase separation technique that allows to differentiation of ions with the same *m/z* values but different molecular structures (isomers). Isomeric separation of ions by ion mobility is performed based on their size, shape, and charge state differences.

Several ion sources used for the ionization of the molecules can be interfaced with MS. Amongst these sources one can find electrospray ionization (ESI), atmospheric pressure chemical ionization (APCI), atmospheric pressure photoionization (APPI) and matrix-assisted laser desorption/ionization (MALDI). The ionization sources that are widely used for biological samples include MALDI and ESI. MALDI source is used for mass spectrometry imaging to analyse chemical distributions of for instance 3D organ models, organoids (Kogler et al., 2023). Since MALDI requires sample preparation processes, it could not be used to analyse cell excretes from OOC in real-time fashion. ESI is the commonly used ionization method in MS when studying the excretes of cells from OOC, in either the off–or on-line mode. Therefore, taking the sensitivity, specificity, and structural identification potential of ESI-MS into account, its hyphenation to OOC devices is potentially offering online and real-time observation of cell behavior.

## Hyphenation of organ-on-a-chip and mass spectrometry

MS as a sensitive and high-throughput technique can provide molecularly specific information. This analytical tool also can detect short-lived reaction intermediates or labile metabolites (Wu et al., 2016). Various research groups have tried to couple OOC and MS, however, there is no established methodology yet. The failures so far are mainly due to the decreased flexibility, clogging, and incompatibility of the solvents when the two systems are connected. This results from the oxygenated medium, consisting of salts and serum that can cause interferences in the MS (Wink et al., 2018; Leipert and Tholey, 2019). Hence, to analyze the biological content of OOC, which contains a complex mixture of components, sample pre-treatment (e.g., purification, extraction, preconcentration) before ionization for MS analysis is essential (Mao et al., 2018). To this end, various pre-treatment techniques and chip designs naming electrophoresis, solid-phase extraction, liquid chromatography, and droplet-based chips have been developed to bridge OOC with MS. Table 2 is summary comparison of these techniques.

**TABLE 2:** Organ-on-a-chip hyphenation techniques to mass spectrometry.

Hyphenation methods	Advantages	Shortcomings
**Capillary electrophoresis**	High speed performance	Limited loading capacity
	High separation efficiency using very low sample volume	High detection limit
	Separation of wide mass range (large and small molecules)	Requiring sample pre-treatment
**Electromembrane extraction**	Online sample pre-treatment (removing salts, buffers, and large molecules)	Re-connection of the EME-chip tubing
	Suitable for fast reaction kinetic study	
**Solid-phase extraction**	Simplified sample pre-treatment method	Long analysis time (minutes range)
	High efficiency	High solvents and reagents consumption
	High throughput screening	Offline washing step
		Clogging
		Insufficient temporal resolution
		Limited mass range treatment
**Liquid chromatography**	High sensitivity and selectivity	Requiring large sample volume (conventional LC systems)
	Identification and quantification	Leakage and blockage at different sites of connections
**Chip-based LC**	Low sample volume	Incompatible for biological (complex) samples
	Low reagent consumption	High back pressure
	Low cycle time	
	High throughput and fast analysis	
	Stability, reproducibility, and high sensitivity	
**Droplet-based chips**	Low sample volume (micro- to femtoliters)	Limited mass range treatment
	Single-cell studies	Requiring multi-step sample handling
		Complicated fabrication and sample preparation process

### Electrophoresis–mass spectrometry for organ-on-a-chip analysis

**FIGURE 2:**
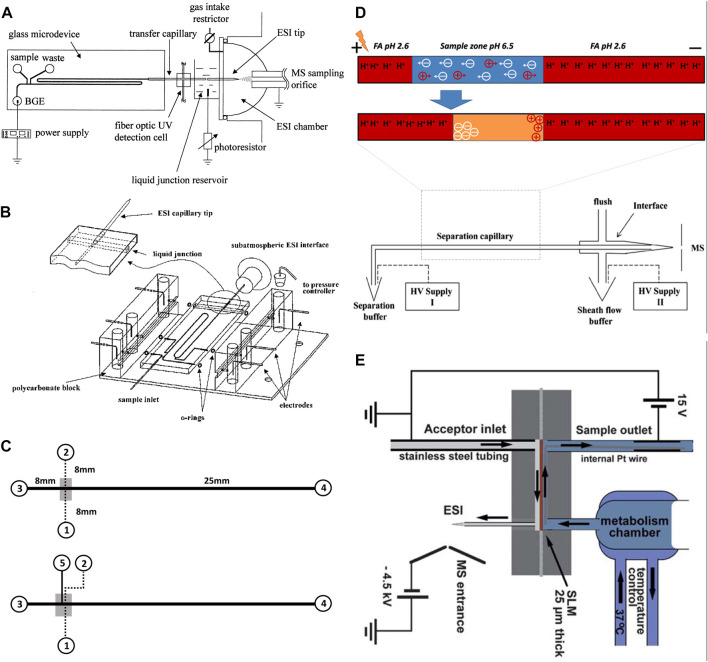
**(A)** Diagram of the chip-based capillary electrophoresis with the capillary transfer line interfaced with the subatmospheric electrospray (Adapted with permission from Zhang et al. (Zhang et al., 1999). Copyright 1999 American Chemical Society). **(B)** Diagram of the chip-based capillary electrophoresis with an integrated pneumatic nebulizer (Adapted with permission from Zhang et al. (Zhang et al., 2001). Copyright 2001 American Chemical Society.) **(C)** Diagram of multilayer microfluidic device (top) the filter-capillary electrophoresis (bottom) the concentrator-capillary electrophoresis with a nanoporous membrane (gray area) (Adapted from Long et al. (Long et al., 2006),.). **(D)** Diagram of the integrate dynamic pH junction preconcentration method with capillary electrophoresis. The HV supply II drives the electrokinetic flow to pump the sheath liquid. The potential difference between the inlet (HV supply I) and outlet (HV supply II) drives the capillary separation (Adapted from Sun et al., and Zhu et al. (Sun et al., 2012; Zhu et al., 2014),). **(E)** Schematic illustration of the electro membrane extraction [Adapted from Petersen et al. (Petersen et al., 2015)].

Electrophoresis is a separation technique, in which an electric field is applied in a running buffer that enables the separation of analytes based on their size and charge. Electrophoresis has been widely used as a separation method in microfluidic chips as it offers high efficiency and requires no stationary phase or high pressure (Wu et al., 2008; Chen et al., 2023). The high efficiency is due to the separation of molecules through shorter separation channels in a short time. These features enable fast detection of analytes, particularly short-lived molecules, reduce fabrication costs, and aid in miniaturization. Capillary electrophoresis (CE) is an electrophoresis-based separation method, which is performed in micro or nano fluidic channels through submillimetre sized capillaries. Integrated CE within the microfluidic chips contributes to super high-speed performance and high separation efficiency using very low sample volume (Hamdan et al., 2000). CE has been used for the separation of small molecules (Jacobson et al., 1994; Jacobson et al., 1998) and large molecules like proteins (Mao et al., 2003; Mao et al., 2004). Under the high electric field of microchip CE, the separation mechanism and behavior of small molecules will not be harmed. In addition, protein separation by CE occurs in a running buffer, which is similar to physiological conditions without requiring complex additives. As a result, protein structure and functions are preserved through the separation process. The chip-based CE interfaced with MS has been reported by the Karger team and Ramsey group (Lazar et al., 1999; Zhang et al., 1999; Zhang et al., 2001). The Karger team introduced glass-based microdevices with an external transfer capillary to connect to the electrospray interface of a MS instrument (Figure 2A). In another design, they also introduced an integrated pneumatic nebulizer (Figure 2B), which omitted the need for an external electrospray port (Zhang et al., 1999; Zhang et al., 2001). The latter design reduced the dead volume and enabled on-chip separation and electrospray of peptides and protein digests to MS. For analysis of proteins, Ramsey and co-workers also reported microchip-based electrophoresis-ESI device to interface with MS (Lazar et al., 1999). However, their designs were only suitable for analyzing small volumes of samples and were not suitable for multiple uses. Having limited loading capacity raise a concern regarding the detection limit, especially for biological samples that include a scarce amount of analyte per volume. To address this concern, a pre-concentration step has been employed along with CE separation. A concentrator-CE was introduced by Lin and his team, in which a nano-porous membrane was placed between two layers of polydimethylsiloxane (PDMS) microchannels (Figure 2C) (Long et al., 2006). Even though this device contributed to desalting and concentrating analytes from human plasma, the small size of pores filters out proteins. An alternative approach is to integrate the dynamic pH junction preconcentration method with CE-ESI-MS (Figure 2D) (Sun et al., 2012). In this method, analytes are prepared in a basic buffer and introduced into a capillary with an acidic buffer. Upon application of an electric field, charged analytes migrated through different zones of the capillary to concentrate and separate. This method has been used by multiple groups (Zhu et al., 2014; Chen et al., 2017; Lubeckyj et al., 2017) and proved to be useful for the injection of large volumes and the detection of a large number of proteins and peptides. Nevertheless, this technique requires pre-treatment of samples before injecting them into the capillary, which limits its application for the online analysis required for OOC platforms. Considering the benefits of electrophoresis, new designs based on the same working principle of this technique were developed to address the online analysis shortcoming.

Electromembrane extraction (EME) is another electrokinetic-based miniaturized sample preparation technique for the extraction of molecules in their ionized form from aqueous media (Huang et al., 2017). EME system is composed of a donor phase (metabolic reaction mixture) and an acceptor phase, making it compatible with MS detection. The two phases are separated by a supported liquid membrane (SLM), filled with organic solvent (usually polypropylene with immobilized 2-nitrophenyl octyl ether). To drive ionized analytes from one phase to the other, an electric field is applied across the membrane (Pedersen-Bjergaard et al., 2017; Li et al., 2022). Petersen *et al.*, (Petersen et al., 2015), experimented with an EME-chip made of polymethyl methacrylate (PMMA) for real-time drug monitoring by ESI-MS (Figure 2E). During their experiment a reaction mixture was continuously perfused by a syringe pump, making contact with the SLM inside the EME-chip. On the other side, the acceptor phase with an organic solvent was also pumped continuously. The electric potential was applied across the SLM by a direct current power supply through small platinum wires, located in both the donor and acceptor phases (Petersen et al., 2015). Once the analytes reach the acceptor phase, they are transferred to MS for detection. The main advantages of EME are the online sample pre-treatment that can remove salts, buffers, and large molecules of the biological samples. In addition, this method enables studying fast reaction kinetics. Nevertheless, one major disadvantage of this design is that before each metabolic experiment, the EME-chip needs to be re-connected with tubing on the acceptor side. Abdossalami *et al.*, (Asl et al., 2015), and Baharfar *et al.*, (Baharfar et al., 2017), used on-chip EME coupled with high-performance liquid chromatography (HPLC) for the enhancement of extraction efficiency. Similar to Petersen *et al.*, these two research groups used PMMA chips, with a SLM made out of a polypropylene sheet, and two platinum electrodes integrated with the donor and acceptor phases (Asl et al., 2015; Baharfar et al., 2017). The experiments conducted by Petersen *et al.*, Abdossalami *et al.*, and Baharfar *et al.*, demonstrated it was possible to concentrate analytes and analyze them with MS. Nevertheless, a persisting drawback of this method is the discrimination of large biomolecules, which could be resolved by implementing specialized SLMs. The electrophoretic separation method directly connected to MS has also been extensively used for cell analysis as reviewed before (Zhou et al., 2019). However, these studies require pre-treatment of samples before performing CE-MS analysis. While online analysis of OOC content requires a direct connection of OOC to MS without stalling the process. This can be a possible future direction for scientists to develop an OOC-EME/CE-MS system for online analysis of OOC, a possibility combined with other online pre-treatment methods such as solid phase extraction.

### Solid-phase extraction–mass spectrometry for organ-on-a-chip analysis

**FIGURE 3:**
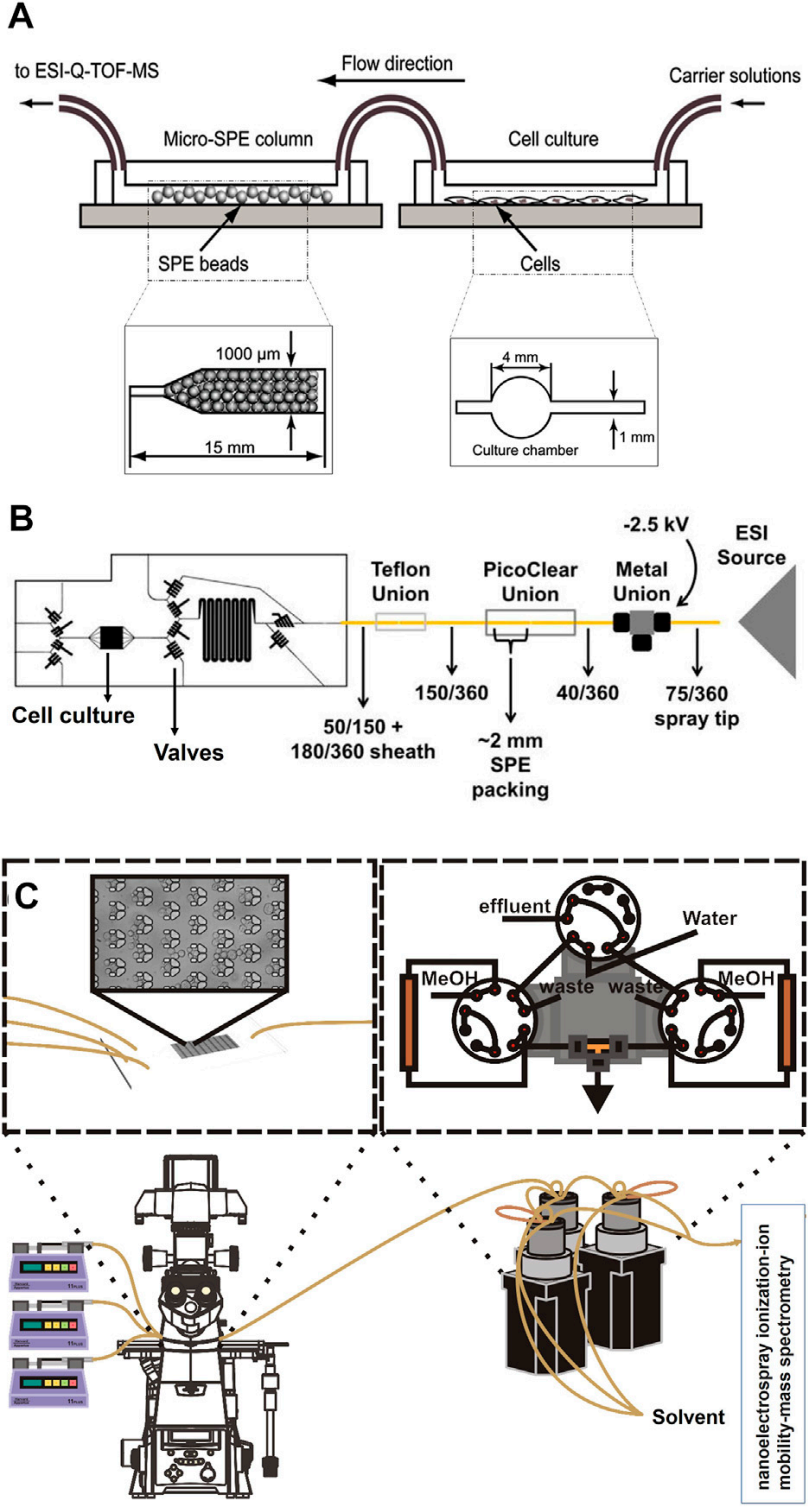
**(A)** Schematic of integrated chip-based solid phase extraction placed between organ-on-a-chip and mass spectrometry with the diagram of the cell culture channel and the narrow-ended microchannel of micro-SPE column (Adapted with permission from Gao et al. (Gao et al., 2010). Copyright 2010 American Chemical Society). **(B)** Schematic of the PDMS-based chip consisted of a cell cultivation chamber and series of the valve to control the on-chip injection loop, which is connected to the SPE column and followed by a spray tip to interface with electrospray ionization source (Adapted from Dugan et al. (Dugan et al., 2017),). **(C)** Schematic of the multi-trap nanophysiometer placed on an inverted fluorescence microscope and connected to solid phase extraction using pumps to control the flow. The setup is continuously analyzed by interfacing with the nanoelectrospray ionization source [Adapted from Marasco et al. (Marasco et al., 2015)].

SPE is another method used by various research groups for the sample pre-treatment before MS analysis. Generally, SPE is used for the extraction and concentration of analytes and purification of interfaces from analytical samples from complex matrices such as urine, blood, animal tissue homogenate extracts, and soil (Gao et al., 2010; Płotka-Wasylka et al., 2016). It is a solid-liquid separation method, which relies on affinity differences among compounds present in a liquid mixture to a solid phase in order to separate and extract. The widely used high throughput SPE systems coupled with MS is mainly relying on an integrated autosampler for the injection of samples from multiwell plates as reviewed before (Luo et al., 2022). However, such a system does not enable an online analysis of OOC complex content. Jin-Ming Lin and his team introduced a novel method to integrate chip-based SPE between OOC and MS junction for online analysis of cultured cells (Gao et al., 2010). In their first design, they fabricated a microfluidic chip made from PDMS with two separate parts, which were connected via polyethylene tubes. The first part of the chip is used for the cultivation of cells and the second part is packed with polymeric SPE beads of 45 µm size (Figure 3A). The outlet of the second part is directly connected to ESI-MS. Before MS analysis, the wash solution of 5% ethanol water was injected into the beads for the removal of unbound materials, proteins, and salts. Subsequently, the purified sample could migrate to the ESI source via fused silica capillary. Using this platform, they could desalt and concentrate the metabolites of interest to study vitamin E metabolism by human lung epithelial cells. The highlight of their design was packing SPE beads in straight microchannels with narrow ends to trap the beads. In addition, this device offers a simplified sample pre-treatment method with high efficiency and potential for high throughput screening of cellular drug metabolism. This group used the same pre-treatment approach for online monitoring of more complex cellular assays by changing the design of the first part of their chip. As such that they could study drug permeability by fabricating two channeled chips separated by a semipermeable membrane to culture cells (Gao et al., 2013). In another study, they performed high throughput drug screening by designing a micro-scale gradient generator connected to a micro-sized cell culture chamber followed by an online SPE chip and MS analysis (Gao et al., 2012). Lin and his team also reported three section chips for cytotoxicity study. The first section of their chip encapsulated human liver microsomes that metabolized acetaminophen. The following section included a cell culture chamber that was directly exposed to the products of acetaminophen. The third section of their chip consisted of a micro SPE column for desalting and concentrating analytes just before direct injection and online monitoring by MS (Mao et al., 2012). Further work has been performed by this work with the same pre-treatment strategy to study cell-cell communications (Mao et al., 2013). Even though this design has proven to be applicable for multiple types of cellular assays, it bears a few drawbacks. This pre-treatment method has a relatively long analysis time (<10 min), which might not be ideal for real-time analysis of fast reactions and cell responses. In addition, this system consumes high volumes of solvents and reagents. On top of all, it requires an offline washing step, which raises concerns regarding the complexity of experiments and the online nature of the system. Dugan *et al.* designed a chip with the feasibility of performing an online washing step. They fabricated a multilayer PDMS chip, which consisted of a cell cultivation chamber and a series of valves (Figure 3B) (Dugan et al., 2017). The operation of valves was similar to a six-port valve and enabled the controlling of an on-chip injection loop, placed right after the cell chamber. The injection loop was connected to a PicoClear union, which was packed with 20 μm sized beads to form a ∼1-mm long SPE column. The other end of the column was directly connected to a spray tip to electrospray the analytes into MS. The designed valve system enabled the on-chip washing step without disconnecting the tubing for offline injections. Even though this design enabled online desalting and concentrating to analyze cell secretion by MS, it still bears the similar drawback of long analysis time (30 min). This concern was addressed by Marasco *et al.*, by constructing a microfluidic bioreactor coupled online to a SPE desalter and MS detector for near real-time analysis of cocaine metabolism by T-cells (Figure 3C) (Marasco et al., 2015). In this design, a low temporal resolution of 9 min was achieved by using an online desalter comprised of two C18 columns, three valves, and two sample loops. One main concern about this platform was about clogging of used filters by cells. This is because their microfluidic bioreactor (multi-trap nanophysiometer) was made from U-shaped traps to study unattached cells. Regardless of multiple advances in the application of online SPE for sample pre-treatment of OOC content before MS analysis, this system still suffers from sufficient temporal resolution, the possibility of clogging by large molecules, and the inability to analyze a wide range of analytes (e.g., proteins).

### Liquid chromatography-mass spectrometry for organ-on-a-chip analysis

Liquid chromatography is the commonly used approach for sample separation before MS. Unlike SPE, which is mainly used for selective sample purification and extraction, LC enables separation of a sample into its individual compounds based on the chemical or physical interaction of compounds with stationary and mobile phases. The conventional LC systems with a large internal diameter of 1–2.1 mm are not the ideal approach for bridging OOC with MS. This is due to the large internal diameter of such columns that requires a large sample volume. This condition does not match the working criteria of microfluidic OOC with a small sample size and low flow rates. The development of narrow-sized LC columns, nano-LC, addresses this issue. Nano-LC-MS, with an inner diameter as low as 75 μm, has been widely used for proteomic and metabolomic studies as reviewed before (Shan and Jones, 2022). This technique offers improved sensitivity, lower flow rate, and lesser injection volume compared to conventional LC systems. However, the relatively low flow rate applied in OOC systems requires implementing concentrators in conjunction with multi-port valves of nano-LC systems. Multi-valve connections increase the possibility of flow leakage and blockage at different sites of connections. This is especially true for packed nano-LC columns. The size of packed particles in nano-LC can go down to 2 μm, which results in improved chromatography. However, lowering particle size comes with the cost of high back pressure and frictional heating effects. This is not an ideal approach for online analysis of OOC content. An alternative to packed nano-LC columns is open tubular LC columns with a much smaller inner diameter of 5–10 µm (Lin et al., 2020). Open tubular LC columns have proven to improve sensitivity, chromatography results, and back pressure concerns (Vehus et al., 2016), however, they have not been widely used for omics studies and not at all used as the interface of OOC and MS. This might be due to columns poor robustness and reproducibility (Bell, 2021). As a result of technological development, chip-based LC platforms appear to address the aforementioned shortcomings. Chip-based LC (Chip-LC) is a miniaturized LC system with its components integrated into a micro-sized chip. This design enables hyphenation with a micro-sized OOC from one side and a MS detector from the other end. Interfacing chip-LC to MS could be done either by integrating an electrospray emitter on the same chip or by tubing connection to the ESI source of a MS instrument (Oedit et al., 2015). As reviewed before (Tsunoda, 2022), chip-LC technology requires low sample volume, reduces reagent consumption and total cycle time (due to its miniaturized nature), and enables high throughput as well as fast analysis. Yin *et al.*, (Yin et al., 2005), used Chip-LC-ESI-MS and developed a microchip integration system on a single-chip device. They established the connection between the two components by laminating the polyimide field with laser-ablated channels, ports, and frit structures. The design contained both enrichment and separation columns, which were packed by the use of conventional reversed-phase chromatography particles. A special face-seal rotary valve was used to switch between sample loading and separation (Yin et al., 2005). Overall, such systems performed well on stability, reproducibility, and sensitivity to identify peptides at low abundance. However, as already known, LC-MS analytical tools are not suitable for biological samples, due to their complexity and the thousands of different compounds within a biological specimen, as well as the high abundance of matrix components that can interfere with the LC-MS analysis. The reason behind this is that complete separation is not possible for complex samples, but rather separation by the grouping of compounds would be made. Hence, one should always keep in mind that purification of the sample is needed before LC-MS analysis by for instance SPE integration. In addition, high back pressure is an inevitable concern when working with micro-scaled fluidic systems.

### Droplet-based chips–mass spectrometry

**FIGURE 4:**
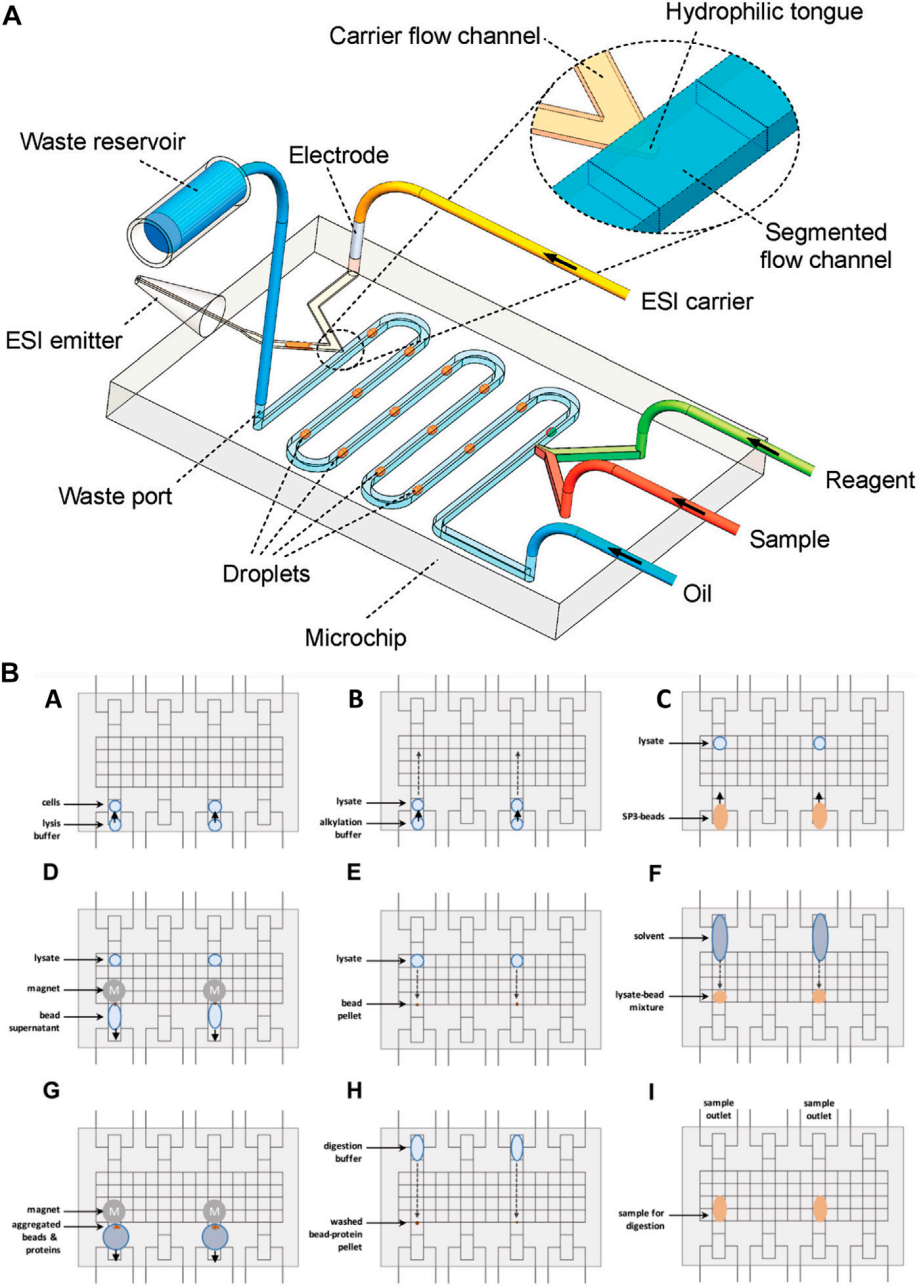
**(A)** Schematic diagram of water and oil droplet-based analysis system interfaced with ESI-MS detection (not to scale) (Adapted with permission from Zhu et al. (Zhu and Fang, 2010). Copyright 2010 American Chemical Society). **(B)** Schematic of the digital microfluidic system for droplet-based sample preparation. **(A)** The reservoir and transfer electrodes are actuated to directly load cell suspension. In the middle of the reservoir, the sample is loaded towards the transfer electrode. To move the droplet into the transfer electrode, the reservoir electrode is deactivated. **(B)** The same principle is used to add a buffer. **(C)** Following buffer loading, the solid-phase-enhanced sample preparation bead mixture is loaded. **(E)** After moving the lysate into the bead pellet, the solution is mixed. **(F)** The solvent is added to induce protein bead aggregation. **(G)** The supernatant is removed upon extracting the beads. **(H)** The bead pellet is mixed with the digestion buffer. **(I)** For 8 h the samples were incubated. Aspirated samples were analyzed by LC-MS [Adapted from Leipert et al. (Leipert and Tholey, 2019)].

Droplet-based microfluidics has been widely used for various biomedical applications. This system enables the performance of cellular assays and chemical reactions in micro-to femtoliters of volumes (Sohrabi et al., 2020; Amirifar et al., 2022). Droplet-based microfluidics has been interfaced with ESI-MS for fast, sensitive, and selective analysis of single-cell studies (Zhang et al., 2016) proteomics (Su et al., 2013) and peptide tracing (Kelly et al., 2009; Pei et al., 2009; Zhu and Fang, 2010). Zhu *et al.*, (Zhu and Fang, 2010), presented an integrated droplet system for ESI-MS sampling. The system aimed the minimalization the interferences of the matrix, by separating the water and oil base droplets containing solute analytes by two-phase flow methods. Thus, after the separation, the droplets are brought to the aqueous phase of the ESI and then further detected by ESI-MS (Figure 4A). The separation of droplets is an example of the enrichment of samples, improving the selectivity and sensitivity analysis. The research group of Zhang *et al.*, also used a droplet-based method, however, they used it to extract the cellular compounds of a single cell for ESI-MS analysis (Zhang et al., 2016). The mechanism is similar to the one used by Zhu *et al.* The target cell was wrapped around by a droplet, containing extraction solvent. After an incubation time, the cell content was extracted to the droplet, which was sucked and dried in the ESI emitter. The dried molecules re-dissolved in an organic solvent, which was then electro-sprayed and detected by MS. Their approach showed that it could selectively acquire and detect the cellular components of a single cell without the interference of other components of the matrix. Nevertheless, the method of Zhang *et al.*, could not detect and extract ATP, ADP, and AMP, due to the high possibility of contaminating the MS instrument with the cytoplasm. The use of droplet-based sample preparation for OOC platforms can be beneficial for the selective analysis of analytes by MS. However, this method does not provide a full range of molecules and requires multi-step sample handling. To improve this method a sample pre-treatment operation must be integrated, such as SPE, for desalting and chemical derivation for it to be used for direct analysis of real biological samples. Another droplet-based manipulation system used for proteomic sample preparation is digital microfluidics (Vitorino et al., 2021). Using this technique, microdroplets are moved, mixed, reacted, and analyzed on a surface with insulated electrodes (Figure 4B). Leipert *et al.*, (Leipert and Tholey, 2019), addressed the mentioned limitations by developing a cell lysing protocol using a digital microfluidic platform for proteome study. On this platform, multiple steps of cell lysis, protein extraction, and protein digestion are performed on a single digital chip. The chip included magnetic beads used as solid phase enhanced sample preparation and clean up. Upon detergent removal, the supernatant was aspirated and pipetted to a LC glass micro insert for LC-MS analysis. This technology enabled the identification of the Jurkat T cells protein profile. However, the complicated fabrication and sample preparation process hinders its application for OOC-MS studies, and it still requires further optimizations for real-time analysis of cellular mechanisms.

## Conclusion

This review looked into connecting OOC to different analytical techniques either on- or off-chip to evaluate and analyse biological content of mimetic tissue model of the OOC, focusing on the coupling of OOC to MS. With the ability of OOCs to mimic human physiology *in vivo*, they open another door to a new generation of research. More particularly, the invention of OOCs brings pharmacological research to a level at which “patient-based” studies within the context of personalized medicine and drug tolerance testing can be performed. Nonetheless, the OOCs approach suffers from the absence of well-validated, fast, and universal analytical detection technologies. Mass spectrometry on the bases of its nature is expected to be a suitable detection and monitoring technology. Yet, its hyphenation of OOC is highly hampered by the lack of fast, accurate, and universal sample pre-treatment technologies. Over the years, several attempts have been made to hyphenate OOC with MS for online and real-time analysis of 3D microcellular cultures. The aim of these approaches has been rapid, precise, and sensitive analysis of cellular mechanisms with minimal chance of outside contamination. Solid phase extraction, electrophoresis-based separations, and liquid chromatography have proven to offer multiple advantages for sample handling in the interface of OOC and MS. Albeit substantial progresses to bridge OOC and MS, there is no concrete solution for online analysis of complex OOC content with MS. Further research focusing on combinatorial approaches that relies on multiple level of extraction and purification could address optimum sample treatment criteria for low flow rate OOC platforms.
